# The pH partition theory predicts the accumulation and toxicity of doxorubicin in normal and low-pH-adapted cells

**DOI:** 10.1038/sj.bjc.6690134

**Published:** 1999-02

**Authors:** L E Gerweck, S V Kozin, S J Stocks

**Affiliations:** Department of Radiation Oncology, Massachusetts General Hospital, Boston, MA, 02114, USA

**Keywords:** pH, pH gradient, doxorubicin, cellular toxicity, cellular accumulation

## Abstract

The accumulation and toxicity of the weak base doxorubicin has been investigated as a function of extracellular pH, intracellular pH and the cellular pH gradient in cells previously cultured under normal (pH 7.4) and low-pH (6.8) conditions. Low-pH-adapted cells exhibit transmembrane pH gradients which substantially differ from normal cells at the same extracellular pH. No relationship was obtained between intracellular pH and the uptake or toxicity of doxorubicin in the two cell types. In contrast, doxorubicin accumulation and toxicity increased with increasing extracellular pH in both normal and low-pH-adapted cells. However, at the same extracellular pH, drug cytotoxicity was more pronounced in normal than in low-pH-adapted cells. The difference in doxorubicin accumulation and cytotoxicity at the same extracellular pH was found to be dependent on the difference in the transmembrane pH gradient of the two cell types. As the cellular pH gradient differs between tumour and normal tissue, this observation suggests a basis for enhancing cellular drug uptake in either tissue type. © 1999 Cancer Research Campaign


*British Journal of Cancer* (1999) **79**(5/6), 838–842
© 1999 Cancer Research Campaign
Article no. bjoc.1998.0134

### The pH partition theory predicts the accumulation and toxicity of doxorubicin in normal and low-pH-adapted cells

**LE Gerweck, SV Kozin and SJ Stocks**

Department of Radiation Oncology, Massachusetts General Hospital, Boston, MA 02114, USA

**Summary** The accumulation and toxicity of the weak base doxorubicin has been investigated as a function of extracellular pH, intracellular pH and the cellular pH gradient in cells previously cultured under normal (pH 7.4) and low-pH (6.8) conditions. Low-pH-adapted cells exhibit transmembrane pH gradients which substantially differ from normal cells at the same extracellular pH. No relationship was obtained between intracellular pH and the uptake or toxicity of doxorubicin in the two cell types. In contrast, doxorubicin accumulation and toxicity increased with increasing extracellular pH in both normal and low-pH-adapted cells. However, at the same extracellular pH, drug cytotoxicity was more pronounced in normal than in low-pH-adapted cells. The difference in doxorubicin accumulation and cytotoxicity at the same extracellular pH was found to be dependent on the difference in the transmembrane pH gradient of the two cell types. As the cellular pH gradient differs between tumour and normal tissue, this observation suggests a basis for enhancing cellular drug uptake in either tissue type.

**Keywords:** pH; pH gradient; doxorubicin; cellular toxicity; cellular accumulation

The cytotoxicity of doxorubicin is strongly influenced by variation in extracellular pH (Born and Eichholtz-Wirth, 1981). As is the case with other weak electrolytes (Brophy and Sladek, 1983; Dennis et al, 1985; Mikkelsen et al, 1985; Skovsgaard, 1977), this variation in toxicity may be due to a pH-dependent shift in the ratio of the ionized to non-ionized form of this weak base. In its non-ionized form, the lipophilicity of weak electrolytes is increased, thereby enhancing their diffusion through the cell membrane to an intracellular site of action. Extensive investigation of the mechanism of doxorubicin uptake indicates that passive diffusion of the non-ionized form of the drug is the most likely explanation for the pH-dependent modification of cellular drug uptake (and by implication, cytotoxicity), but that carrier-mediated uptake cannot be excluded with the specificity or efficiency of the carrier being dependent on the ionization status of the drug (Skovsgaard, 1977). The toxicity of a number of drugs is sensitive to variation in pH by various mechanisms which are not dependent on ionization-dependent diffusion through the cell membrane (Jähde et al, 1989; Skarsgard et al, 1995). For example, extracellular pH variation substantially modifies the toxicity but not the cellular uptake and accumulation of the actively transported drug melphalan (Skarsgard et al, 1995). Additionally, it has been reported that the cytotoxicity of free doxorubicin is identical to doxorubicin bound to large particles excluded by the cell (Tritton and Yee, 1982). These results suggest an extracellular (membrane) site of doxorubicin action, i.e. with toxicity not being dependent on drug uptake.

To the extent toxicity is dependent upon the intracellular accumulation of doxorubicin, variation in pH and its resultant effect on drug charge and membrane diffusion would affect the drug's intracellular concentration and therefore its toxicity (Eichholtz-Wirth, 1980). In theory, at steady state, the intravesicular concentration of a weak electrolyte which is impermeable in its charged form, but membrane permeable in its uncharged form, is dependent upon the magnitude of the pH gradient across the cell membrane and the drug p*K*a (Roos and Boron, 1981). This has important implications for the differential uptake and potentially the toxicity of weak electrolytes, such as doxorubicin, in tumour and normal tissue. Although the intracellular pH of tumour and normal tissue is similar, the extracellular pH of human tumours is more acidic than normal tissues, giving rise to substantially different cellular pH gradients in these tissues (Vaupel et al, 1989; Gerweck and Seetharaman, 1996).

To evaluate the role of extracellular and intracellular pH and the pH gradient on doxorubicin accumulation and cytotoxicity, studies were conducted on normal and low-pH-adapted Chinese hamster ovary cells in vitro. As previously described, low-pH-adapted cells are resistant to changes in intracellular pH upon reduction of extracellular pH (Kozin and Gerweck, 1998). Therefore, at a particular extracellular pH, different pH gradients are obtained in the two cell types, whereas at a similar intracellular pH the extracellular pH substantially differs.

### MATERIALS AND METHODS

### Cell culture

Chinese hamster ovary cells were cultured and studied in Ham's F-12 medium supplemented with 12% fetal bovine serum plus antibiotics. The medium was buffered with 15 mM Hepes, 10 mM EPPS, plus approximately 3 mM sodium bicarbonate from the serum. Medium pH was adjusted with 1 N hydrochloric acid or 1 N sodium hydroxide at 37°C. Cells were grown as subconfluent monolayers and transferred twice per week. Normal cells were

*Received 29 April 1998*
*Accepted 24 July 1998*

*Correspondence to:* LE Gerweck cultured in medium adjusted to pH 7.4, and acid-adapted cells continuously cultured (> 2 months) in medium adjusted to pH 6.8. During culture, the pH of the medium decreased by approximately 0.2 pH units over 3–4 days in both cell types. The doubling times of the normal and acid-adapted cells were similar, i.e. 13–14 and 14–15 h respectively. All experimental studies were performed on exponentially growing cells.

### Measurement of intracellular pH

Intracellular pH was evaluated by the method originally developed by Waddell and Butler (1959), which is based on the equilibrium distribution of the weak acid [$^{14}$C]DMO ([2-$^{14}$C]5,5-dimethyl-2,4-oxazolidinedione) across the cell membrane. This technique, with some modification, has frequently been used by others and is described in detail elsewhere (Chu and Dewey, 1988; Fellenz and Gerweck, 1988; Kozin and Gerweck, 1998). Briefly, trypsinized cell suspensions (5–10 × $10^5$ cells ml$^{-1}$) were concurrently labelled with [$^3$H]water and [$^{14}$C]DMO or [$^{14}$C]inulin. Twenty to thirty minutes after the adjustment of the extracellular pH ($pH_e$), samples of the suspension were centrifuged through 0.2 ml of silicone oil into 0.06 ml of 0.8 M perchloric acid. Aliquots of the supernatant and perchloric acid cell extracts were removed for determination of total and extracellular water and intracellular and extracellular DMO. These data were used to calculate intracellular pH ($pH_i$) at various $pH_e$ (Kozin and Gerweck, 1998).

### Measurement of cellular doxorubicin

Cell suspensions (5–10 × $10^6$ cells ml$^{-1}$) were prepared in medium at the appropriate pH, brought to 37°C and spiked with doxorubicin (doxorubicin hydrochloride VHA, Irving, TX, USA) also prepared in pH-adjusted medium to yield a final concentration of 10 μg ml$^{-1}$. The suspension was continuously agitated with a reciprocal shaker, and 1-ml samples were periodically removed for up to 100 min. The sample was layered on the top of a two-phase combination of silver nitrate (5.5%, 100 μl, lower phase) (Schwartz, 1973) and silicone oil (200 μl, upper phase) in a polypropylene microcentrifuge tube, and immediately centrifuged. The upper medium layer was sampled for measurement of extracellular doxorubicin. The pellet was resuspended in the silver nitrate solution and extracted for 30 min at 0°C. The silver nitrate extract and medium were further extracted with chloroform/methanol (9:1), dried over nitrogen, and dissolved in 100 μl formate buffer (0.1% ammonium formate, pH 4.0) for high-performance liquid chromatography (HPLC) analysis (Israel et al, 1978). Samples (35–75 μl) were separated on a 150 × 0.9 mm i.d. column containing a $C_{18}$ 5-μm silica particle stationary phase and a mobile phase of 0.1% ammonium formate, pH 4, and acetonitrile (3:1), at a flow rate of 0.6 ml min$^{-1}$. Doxorubicin was quantitated by area under the curve absorbance at 479 nm compared with known concentrations of doxorubicin. Doxorubicin metabolites were not detected. The HPLC measurements yielded the total extracted cellular concentration of doxorubicin and the adhering fluid layer surrounding the cells. The size of the extracellular compartment was determined by analysis of the fraction of total water per cell ([$^3$H]water) and extracellular water ([$^{14}$C]inulin). After adjusting for doxorubicin contained in the adherent extracellular water space, the doxorubicin concentration per unit cell water was calculated.

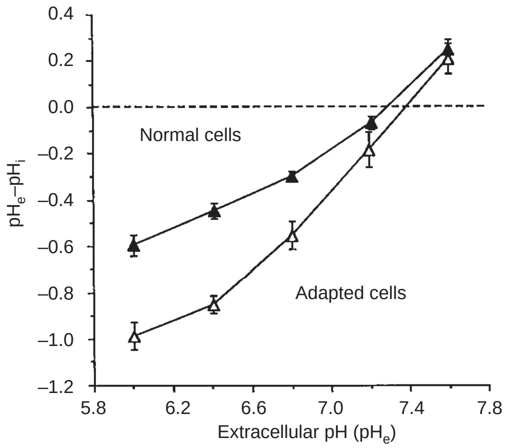

**Figure 1** The difference between the intracellular and extracellular pH ($pH_e$–$pH_i$) is plotted as a function of extracellular pH for normal and low-pH-adapted cells. Each data point is the mean and s.d. of 12 sample assays from three independent experiments. Intracellular pH values were determined at 20–30 min after adjustment of extracellular pH. Confidence intervals of extracellular pH values are ≤ 0.05 units

### Evaluation of drug cytotoxicity

Doxorubicin was freshly diluted in medium at the appropriate pH before use and added to single cell suspensions (2 × $10^5$ cells ml$^{-1}$) at pH 7.4, 6.8 or 6.4 (± 0.05). Cells were incubated for 90 min at 37°C with gentle continuous agitation on a reciprocal shaker.

After drug treatment, the cells were centrifuged, washed twice with drug-free medium and seeded in 25 cm$^2$ plastic flasks to yield 50–200 colonies. Four to six flasks were used for each data point. Medium at pH 7.4 was used for washing and cloning of normal cells, and pH 6.8 medium was used for acid-adapted cells. After incubation, the colonies were stained and counted. Cell survival was calculated as the ratio of the number of colonies divided by the number of cells plated in treatment vs control flasks. In the absence of drug treatment, the plating efficiency was close to 100% independent of the pHe and cell type. The drug enhancement ratio (ER) was calculated as the ratio of drug doses yielding a surviving fraction of 10% in normal cells at pHe 6.8 compared with the drug concentration yielding the same surviving fraction for the various other (pH and cell type) experimental conditions. All survival curve experiments were repeated three times; normal and low-pH-adapted cells were concurrently evaluated in each experiment at the same extracellular pH.

### Calculation of the predicted intracellular drug concentration

The measured changes in drug cytotoxicity as a function of the pH gradient and measured cellular concentration of doxorubicin were compared with the predicted changes in the intracellular (cytoplasmic) concentration of the drug. For doxorubicin, as for a weak base with one ionizing group, the predicted intracellular/extracellular concentration ratio at equilibrium is:

$$C_i/C_e = (1 + 10^{pKa - pH_i})/(1 + 10^{pKa - pH_e}),$$

in which $C_i$ and $C_e$ are the total (charged plus uncharged) intracellular and extracellular drug concentrations respectively (Roos and Boron, 1981). The p$K$a of doxorubicin is approximately 8.2 with remaining p$K$as > 9.5 (Skovsgaard, 1977).

© Cancer Research Campaign 1999 

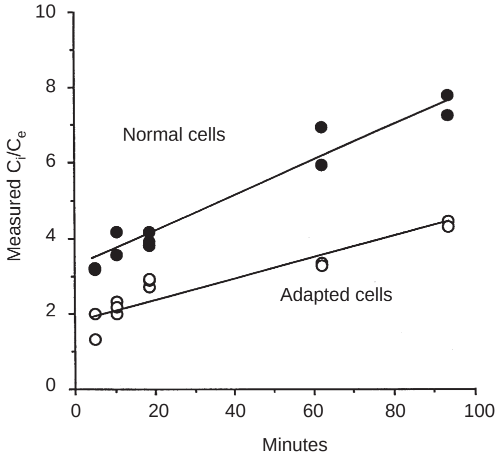

**Figure 2** The measured intracellular to extracellular concentration ratio of doxorubicin ($C_i/C_e$) is plotted as a function of time after doxorubicin addition. The extracellular pH was adjusted to 6.3 approximately 30 min before drug addition. Normal and low-pH-adapted cells were analysed concurrently. One data point per sample from two independent experiments

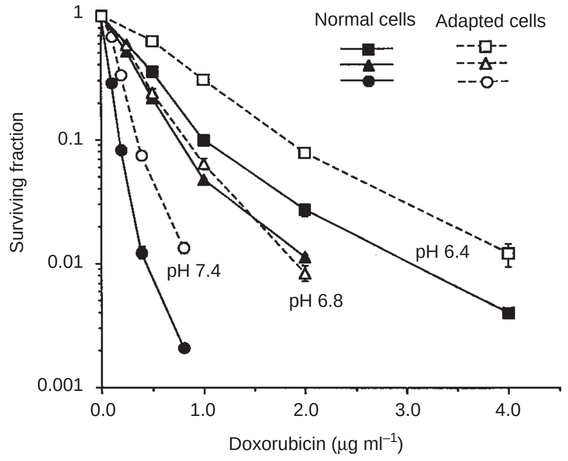

**Figure 4** Doxorubicin cell survival curves were measured at extracellular pH values of 7.4, 6.8 and 6.4 (± 0.05 pH units). The means and standard errors of three separate experiments are shown. For each experiment, normal and low-pH-adapted cells, at the same extracellular pH, were analysed concurrently

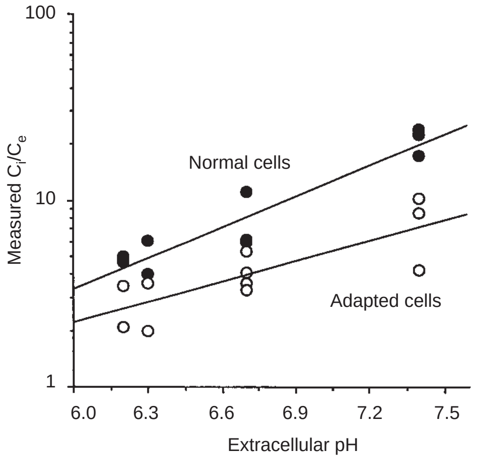

**Figure 3** The measured intracellular to extracellular concentration ratio of doxorubicin is plotted as a function of extracellular pH. The drug concentration ratio was measured 90 min after doxorubicin addition. The results of independent experiments containing two or more pairs of concurrent measurements in normal and low-pH-adapted cells are shown. Extracellular pH values are ± 0.05 pH units

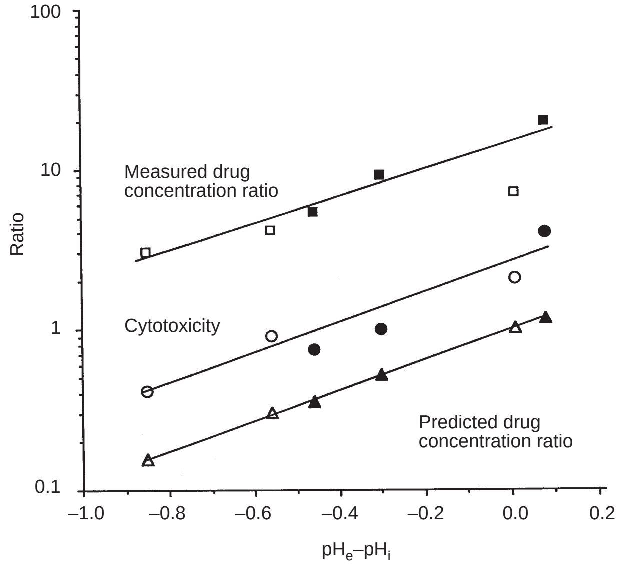

**Figure 5** The measured and predicted intracellular to extracellular concentration ratio of doxorubicin, and drug cytotoxicity enhancement ratio, are plotted as a function of the difference between the intracellular and extracellular pH ($pH_e$–$pH_i$). The predicted concentration ratio assumes no drug binding or metabolism. The measured concentration ratio is for 90 min exposure to doxorubicin. Normal cells are indicated by closed symbols; low-pH-adapted cells by open symbols

### RESULTS

The relationship between the extracellular pH and the difference between the intracellular and extracellular pH for normal and low-pH-adapted cells is shown in Figure 1. At extracellular pH values > 7.1, the magnitude of the pH gradient is similar in both cell types, however at pH values < 7 the intracellular pH of cells adapted to low-pH conditions is relatively resistant to extracellular pH changes and the pH gradient difference continuously increases. At an extracellular pH in the range of 6.0–6.4, the cellular pH gradient in adapted cells is approximately 0.4 units greater than in their unadapted counterparts.

Changes in the intracellular to extracellular drug concentration ratio at pH 6.3 as a function of time after doxorubicin addition is shown in Figure 2. In both cell types, the cellular drug concentration linearly increases with drug exposure time. Consistent with the pH partition theory, in normal cells for which the intracellular pH is less alkaline compared with the extracellular compartment, a greater fraction of the weak base is ionized and therefore accumulated in the intracellular compartment. A similar time-dependent linear increase in cellular drug concentration was observed at all extracellular pH values examined. From experiments similar to those shown in Figure 2, the intracellular to extracellular drug concentration ratio was determined at 90 min, i.e. identical to the drug exposure time for the toxicity assays. The results are shown in Figure 3. Regardless of the extracellular pH, the intracellular doxorubicin concentration is consistently higher in normal than

© Cancer Research Campaign 1999

adapted cells, although the measured magnitude of this difference varied slightly from experiment to experiment.

The lethal response of cells to doxorubicin at extracellular pH of values of 6.4, 6.8 and 7.4 is shown in Figure 4. For both normal (solid curves) and low-pH-adapted cells (dashed curves), pH variation markedly influences toxicity. For example, for a 90-min exposure to 0.5 mg $ml^{-1}$ doxorubicin, the surviving fraction of normal cells was reduced by a factor of approximately 50 at an extracellular pH 7.4 vs 6.4. At an extracellular pH of 6.4 and 7.4, low-pH-adapted cells were substantially more resistant to doxorubicin than normal cells.

The drug cytotoxicity enhancement ratio is plotted as a function of the difference between the intracellular and extracellular pH ($pH_e - pH_i$) in Figure 5. Normal cells are indicated by closed circles and adapted cells by open circles. The data for both cell types are fitted well by a single curve, indicating that, for both cell types, the cellular pH gradient is the determinant of variable cytotoxicity.

Two additional curves are shown in Figure 5. The lower curve (triangles) is the expected intracellular/extracellular drug concentration ratio ($C_i/C_e$) of doxorubicin at equilibrium if the ionized form of the drug is membrane impermeable, the non-ionized form is permeable and the drug is neither sequestered or metabolized. These data are calculated from the equation above. The slope of this curve (0.94 ± 0.12) is identical to the slope of the measured drug enhancement ratio curve (0.95 ± 0.15).

The upper curve of Figure 5 is the measured intracellular/extracellular doxorubicin concentration ratio after 90 min drug exposure. As might be expected (see Discussion section), at a particular pH gradient, the values of the measured and predicted drug concentration ratios differ. Similar to the results observed with drug toxicity, the data showing the relationship between the pH gradient and measured intracellular doxorubicin concentration both for normal and low-pH-adapted cells are fitted well by a single curve. The slope of this curve (0.71 ± 0.20) does not significantly differ from the toxicity and predicted drug concentration ratio curves.

### DISCUSSION AND SUMMARY

The results of this study clearly show that doxorubicin accumulation and toxicity increase with increasing medium pH. pH variation may exert its drug modifying effect intracellularly, extracellularly or via change in the cellular pH gradient. The pH-dependent effect is clearly not related to changes in intracellular pH. For both cell types and all pH conditions, the relationship between pHi and the measured drug accumulation or toxicity enhancement ratio is poor ($r^2 = 0.02$ and 0.19 respectively). Whether pH modification of drug uptake and toxicity is dependent upon the change in extracellular pH or the pH gradient is less straightforward. This is because a change in extracellular pH results in an associated change in the pH gradient, especially in normal cells.

An analysis of the results obtained from both normal and low-pH-adapted cells helps resolve this question. As seen in Figures 2 and 3, both the rate of accumulation and total accumulation at 90 min clearly differs in normal vs low-pH-adapted cells at the same extracellular pH. Additionally, at a $pH_e$ of 6.4, in which the most pronounced gradient difference between normal and low-pH-adapted cells occurs, normal cells are substantially more sensitive to doxorubicin than their low-pH-adapted counterparts. This indicates that the pH gradient and not extracellular pH is the major determinant of doxorubicin uptake. The relationship between the predicted and observed results are not, however, quantitatively precise. The measured magnitude of the gradient difference in normal vs adapted cells is greater at $pH_e$ 6.4 > 6.8 > 7.4. At these extracellular values, the pH gradient hypothesis predicts that normal cells will be more sensitive (and have greater drug uptake) than adapted cells, and this is observed. However, the measured difference in sensitivity in normal vs adapted cells is less than the predicted difference at a pHe of 6.8, and greater than is predicted at a $pH_e$ of 7.4. Whether this inconsistency is due to a lack of precision in measuring the magnitude of the gradient, cytotoxicity or other variables is unclear. However, it does not appear to be due to changes in intrinsic properties of adapted cells because of prolonged culture at low pH (e.g. drug membrane permeability).

It is also of note that for a particular pH gradient value (Figure 5) the measured doxorubicin accumulation (intracellular/extracellular concentration ratio) is substantially greater than the predicted ratio. This is not unexpected because doxorubicin is readily bound and sequestered at various intracellular sites and compartments, especially DNA and lysosomes (Noël et al, 1978; Durand, 1981), and continually accumulates during the 90-min duration of drug exposure. However, because the quantity of drug which accumulates intracellularly is proportional to the quantity of drug which gains access to the intracellular compartment, the numerical value of the measured ratios would change proportionally, leaving the slopes of the curves vs pH gradient unchanged as is observed.

To summarize, the relationship between changes in pH and the uptake and toxicity of doxorubicin is well predicted by the pH partition theory in normal and low-pH-adapted cells. These results further support the suggestion that the design and utilization of weak acid or bases with appropriate p*K*as may be expected to enhance cellular uptake of available drug in tumour or normal tissue because of the naturally occurring cellular pH gradient differences in these tissues (Gerweck and Seetharaman, 1996; Kozin and Gerweck, 1998).

### REFERENCES

Born R and Eicholtz-Wirth H (1981) Effect of different physiological conditions on the action of adriamycin on chinese hamster cells in vitro. *Br J Cancer* **44**: 241–246

Brophy GT and Sladek NE (1983) Influence of pH on the cytotoxic activity of chlorambucil. *Biochem Pharma* **32**: 79–84

Chu GL and Dewey WC (1988) The role of low intracellular or extracellular pH in sensitization to hyperthermia. *Radiat Res* **114**: 154–167

Dennis MF, Stratford MRL, Wardman P and Watts ME (1985) Cellular uptake of misonidazole and analogues with acidic or basic functions. *Int J Radiat Biol* **47**: 629–643

Durand RF (1981) Flow cytometry studies of intracellular adriamycin in multicell spheroids in vitro. *Cancer Res* **41**: 3495–3498

Eicholtz-Wirth H (1980) Dependence of the cytostatic effect of adriamycin on drug concentration and exposure time in vitro. *Br J Cancer* **41**: 886–891

Fellenz MP and Gerweck LE (1988) Influence of extracellular pH on intracellular pH and cell energy status: relationship to hyperthermic sensitivity. *Radiat Res* **116**: 305–312

Gerweck LE and Seetharaman K (1996) Cellular pH gradient in tumor versus normal tissue: potential exploitation for the treatment of cancer. *Cancer Res* **56**: 1194–1198

Israel M, Pegg WJ, Wilkinson PM and Garnick MC (1978) Liquid chromatographic analysis of adriamycin and metabolites in biological fluids. *J Liquid Chromatogr* **1**: 759–809

Jahde E, Glusenkamp K, Klunder I, Hulser DF, Tietze L and Rajewsky MF (1989) Hydrogen ion-mediated enhancement of cytotoxicity of bis-chloroethylating drugs in rat mammary carcinoma cells in vitro. *Cancer Res* **49**: 2965–2972

Kozin SV and Gerweck LE (1998) Cytotoxicity of weak electrolytes after the adaptation of cells to low pH: role of the transmembrane pH gradient. *Br J Cancer* **77**: 1580–1585

*© Cancer Research Campaign 1999*

Mikkelsen RB, Asher C and Hicks ST (1985) Extracellular pH transmembrane distribution and cytoxicity of chlorambucil. *Biochem Pharma* **34**: 2531–2534
Noel G, Peterson C, Trouet A and Tulkens P (1978) Uptake and subcellular localization of daunorubicin and adriamycin in cultured fibroblasts. *Eur J Cancer* **14**: 363–368
Roos A and Boron WF (1981) Intracellular pH. *Physiol Rev* **61**: 296–434
Schwartz HS (1973) A fluorometric assay for daunomycin and adriamycin in animal tissues. *Biochem Med* **7**: 396–404
Skarsgard LD, Skwarchuk MK, Vinczan A, Kristl J and Chaplin DJ (1995) The cytotoxicity of melphalan and its relationship to pH, hypoxia and drug uptake. *Anticancer Res* **15**: 219–224
Skovsgaard T (1977) Transport and binding of daunorubicin, adriamycin, and rubidazone in Ehrlich ascites tumour cells. *Biochem Pharma* **26**: 215–222
Tritton TR and Yee G (1982) The anticancer agent adriamycin can be actively cytotoxic without entering cells. *Science* **217**: 248–250
Vaupel P, Kallinowski F and Okunieff P (1989) Blood flow, oxygen and nutrient supply, and metabolic microenvironment of human tumors: a review. *Cancer Res* **49**: 6449–6465
Waddell WJ and Butler TC (1959) Calculation of intracellular pH from the distribution of 5,5-dimethyl-2,4-oxazolidinedione (DMO). Application to skeletal muscle of the dog. *J Clin Invest* **38**: 720–729

*© Cancer Research Campaign 1999*
